# Exploring the Associations between Happiness, Life-satisfaction, Anxiety, and Emotional Regulation among Adults during the Early Stage of the COVID-19 Pandemic in Russia

**DOI:** 10.11621/pir.2023.0106

**Published:** 2023-03-30

**Authors:** Dmitriy S. Kornienko, Natalya A. Rudnova

**Affiliations:** a Russian Presidential Academy of National Economy & Public Administration, Moscow, Russia; b Psychological Institute of the Russian Academy of Education, Moscow, Russia

**Keywords:** Happiness, Life-satisfaction, COVID-19, perceived stress, anxiety, emotional regulation, cognitive reappraisal

## Abstract

**Background:**

The COVID-19 pandemic is not only a world health crisis, but also an ordeal for people’s mental health and psychological well-being. The period of the COVID-19 lockdown has changed everyday life and increased anxiety, fears, and stress from habitual activities such as meetings, shopping, and the use of public transport. As the worry and nervousness increase, they threaten the cognitive (Life-satisfaction) and emotional (Happiness) components of well-being. Emotional regulation strategies are a mechanism to cope with the threat.

**Objective:**

This study assessed the impact of anxiety, perceived stress from COVID-19, and emotional regulation strategies on well-being during the first weeks of the lockdown in Russia.

**Design:**

Questionnaire-based surveys were conducted online from March 31 to April 30, 2020. A total of 589 participants (18 to 73 years of age) were recruited. The Subjective Happiness Scale, Satisfaction with Life Scale, Zung’s Self-Rating Anxiety Scale, Emotion Regulation Questionnaire, and Perceived Source of Stress from COVID-19 scales were used.

**Results:**

Among the various sources of stress, only that from restrictions on everyday life impacted well-being. High anxiety, but not perceived stress, decreased the feelings of Happiness and Life-satisfaction. Additionally, emotional regulation strategies played different roles in their impact on well-being: Cognitive reappraisal lowered negative emotions, but emotional suppression increased dissatisfaction with life.

**Conclusion:**

These findings suggest that people’s effective and relevant regulation of their emotions during public health emergencies and ability to avoid losses caused by crisis events, have become urgent needs, requiring the development of psychological interventions to support well-being.

## Introduction

Since December 2019, COVID-19 has spread rapidly all over the world (WHO, 2020). Globally, there were 126,372,442 confirmed cases of COVID-19 and 2,769,696 deaths as of March 28, 2021 (WHO, 2021). The risk of death and the virus’s effects on health have put significant psychological pressure on people all over the world (Duan & Zhu, 2020; Xiao, 2020; Ryaguzova, 2021). The continuous spread of the pandemic and implementation of quarantine measures across the globe have negatively affected people’s mental health. According to the research done in China and Italy, diverse societal groups (medical staff and health workers, patients, students, and the elderly) have reported the negative psychological impact of the pandemic (Bao et al., 2020; Chen et al., 2020; Somma et al., 2020; Yang et al., 2020).

### Perceived stress and the pandemic

The spread of the COVID-19 pandemic provoked stress, fears, and anxiety (Boateng et al., 2021; Ongaro et al., 2021), but these typical reactions led to different consequences (Morosanova, 2021; Zinchenko et al., 2021). They ranged from adopting behaviors to prevent infection with COVID-19, to unrealistic optimism, and negative effects on people’s psychological well-being (Taylor, 2019; Cao et al., 2020; Samokhvalova et al., 2022). More than one-third of U.S. students agreed that they were stressed about the health implications of getting COVID-19 (Cohen et al., 2020). Other studies mentioned that stress symptoms became a long-term burden (Brailovskaia & Margraf, 2020), and led to low sleep quality during the coronavirus outbreak (Zhao et al., 2021b). This perceived stress can be considered one of the significant obstacles to psychological well-being, affecting general happiness and the quality of life. High perceived stress is significantly associated with increased worry, nervousness, apprehension, and somatic symptoms (Zung, 1971; Lee, 2012; Dunstan et al., 2017; Zhao et al., 2021b).

### Happiness and Life-satisfaction during the pandemic

Among other features of well-being and quality of life, Happiness and Life-satisfaction play leading roles. These constructs are inherently separate and describe different elements of well-being. Happiness can be described as the predominance of a positive affect, effective emotional regulation, and the ability to cope with problems; it represents the affective component of well-being (Lyubomirsky et al., 2005). Happiness has correlated negatively with anxiety, depression, and perceived stress during the pandemic among the various social groups (Brivio et al., 2021; Yarrington et al., 2021). Life-satisfaction is the cognitive component of well-being, “referring to a judgmental process in which individuals assess the quality of their lives on the basis of their own unique set of criteria” (Pavot & Diener, 1993). Recent studies show that Life-satisfaction has negative associations with psychological distress, and that the anxiety related to the COVID-19 pandemic reduced Life-satisfaction (Duong, 2021; Shchukina & Shchirman, 2022).

### Well-being enhancement involving emotional regulation strategies

According to some recent studies, people’s levels of Happiness and Life-satisfaction can depend on their strategies for emotional regulation (Ng, 2018; Mahmoodi Kahriz et al., 2020). Effective emotional regulation, in turn, is associated with different psychological benefits and helps people manage many work, educational, and life challenges (Aldinger et al., 2013; Chen et al., 2020). Thus, emotional regulation strategies may be crucial to reducing the harmful effects of stress. The surge in worry and stress during the pandemic reduced well-being, and improved self-regulation may help deal with it.

The process model of emotional regulation includes two main strategies: cognitive reappraisal and expressive suppression (Gross, 2015; Gross & John, 2003). A person’s preference in emotional regulation strategies correlates with differences in levels of well-being, self-esteem, and aggression (Gross & John, 2003; Goldin et al., 2014; Chen et al., 2020; Zhang et al., 2020). Using the maladaptive strategy of emotional suppression correlates with depression and a low valuation of one’s happiness (Mahmoodi Kahriz et al., 2020). It has also been found that the ability to cope with uncontrollable stressful life events and Life-satisfaction are mediated by cognitive reappraisal and emotional suppression (Ng, 2018).

### Anxiety, well-being, and the pandemic

Compared to the pre-pandemic state, anxiety due to the fear of the unknown (size of the pandemic, disinformation about the virus, the fatality of disease, etc.) intensified during the initial weeks of lockdown. Also, the spread of COVID-19 increased depressive symptoms and lowered emotional well-being (Low et al., 2021). Some studies reported an association between emotional suppression and anxiety during the COVID-19 pandemic because avoiding unpleasant things and feelings has paradoxically led to a rise of anxiety and worsening of well-being (Zhao et al. 202a). However, to date, there has been no direct testing of the impact of emotional regulation on the relationship between anxiety and well-being during the COVID-19 lockdown.

### Study objectives

Since recognizing and appreciating the power of emotional regulation in the face of the COVID-19 pandemic is relevant to both research and practical objectives, this study aimed to test the impact of anxiety, perceived stress from COVID-19, and emotional strategies on well-being (Happiness, Life-satisfaction) in the first weeks of the lockdown. Based on previous results (Low et al., 2021; Zhao et al. 2021a), we formulated the following hypotheses: (H1) Anxiety and perceived sources of stress lower Happiness and Life-satisfaction; (H2) Different features of perceived stress have different associations with Happiness and Life-satisfaction; and (H3) Emotional regulation strategies can mediate relationships between the components of well-being and anxiety, reducing the negative impact of anxiety.

## Methods

### Participants

A total of 589 people from three Federal Districts in the Russian Federation (62% from the Volga Federal District; 21% from the Far Eastern Federal District; 7% from the Central Federal District; 12% not specified) were invited to complete a questionnaire (mean age 33.2, SD = 13.03 years, age range 18–73). Among them, 24% were male, 31% were students, and only 5% worked in medical professions. Most participants (92.8%) had at least a university degree or were university students.

### Procedure

Social networks were used to distribute the questionnaire. The participants were asked to report on their health status at the time of the survey. None reported pandemic-related symptoms. All questionnaires were presented to participants in the same order. Every participant in the survey received a report on his or her anxiety, stress level, and regulation strategies after completing the evaluation. Participation in the study was completely voluntary and unpaid.

The data was collected from March 31 to April 30, 2020. Over that time period, Russia’s coronavirus cases grew from 2,337 to 106,498. During this period, to avoid the spread of the coronavirus, the state government restricted foreign flights and travel, prohibited sports and cultural activities, and recommended online forms for education and employment. Throughout the country, the media spread information about the dangers and effects of COVID-19, and rigorous sanitation and health-related measures were implemented and strongly enforced (Plotnikov, 2020).

### Questionnaires

All participants completed the questionnaires, which included Zung’s Self-Rating Anxiety Scale (Zung, 1971); the Emotion Regulation Questionnaire (ERQ) (Gross & John, 2003); a modified Perceived Source of Stress Scale (Wong et al., 2007); the Subjective Happiness Scale (SHS) (Lyubomirsky and Lepper, 1999); and the Satisfaction with Life Scale (SWLS) (Diener et al., 1985).

Zung’s Self-Rating Anxiety Scale (SAS) (Zung, 1971; Mammadova et al., 2012) consists of 20 questions with a four-point Likert-type scale to grade their responses. The SAS has shown good internal consistency, with a Cronbach’s alpha of 0.85 for this study.

The Russian version of the Emotion Regulation Questionnaire (ERQ) (Gross & John, 2003; Pankratova & Kornienko, 2017) was used to measure the two emotional regulation strategies: Cognitive Reappraisal and Emotional Suppression. The ERQ is a 10-item self-reported measure where each item is rated on a 7-point Likert scale. This questionnaire demonstrated a good Cronbach’s alpha: 0.83 for the Cognitive Reappraisal subscale, and 0.72 for the Emotional Suppression one.

The participants’ perceived sources of stress from the COVID-19 outbreak were measured with a 20-item questionnaire that described various situations one might encounter during the pandemic. A 6-point Likert-type scale ranging from 1 (never) to 6 (severe) was used to grade their responses. This questionnaire was based on Wong et al.’s (2007) scale for measuring the perceived source of stress from SARS. For this study, only one modification was made — replacing “SARS” with “COVID-19” — because these two diseases have similar epidemiology, pathogenesis, and clinical characteristics (Liu et al., 2020).

Five scales were measured: 1) Stress from Fear of Infection (*e.g*., “I feel that I might be infected by the COVID-19 virus at any moment”); 2) Stress from Virus Spreading (*e.g.,* “I feel COVID-19 will spread quickly”); 3) Stress from Pandemic Outcomes (*e.g*., “I feel that COVID-19 patients might suffer serious consequences’’); 4) Stress from Everyday Life Restrictions (*e.g*., “I feel COVID-19 restricts my social meetings with my friends”); and 5) Stress from Places and Transport (*e.g.,* “I am afraid to take any public transport”). The Cronbach’s alpha for the scales varied from 0.63 to 0.88 for this study.

The Subjective Happiness Scale (SHS) (Lyubomirsky & Lepper, 1999; Osin & Leontiev, 2020) was used. This scale includes four items on global happiness. The response format is a 7-point Likert scale. For this study Cronbach’s alpha was 0.76.

The judgmental component of subjective well-being was measured using the Satisfaction with Life Scale (SWLS) (Dinner et al., 1985; Osin & Leontiev, 2020). This scale involves five items describing opinions on Life Satisfaction as a whole, using a 7-point Likert scale. Cronbach’s alpha for this scale was 0.84.

### Data analysis

The data were analyzed using a psych package in R environment, IBM SPSS Statistics 20 and PROCESS, a computational plugin for SPSS that conducts mediation, moderation, and conditional process modelling (Hayes, 2013). Pearson’s correlation analysis was used to study the relationships between the variables, and multiple linear regression and mediation analysis was used for testing the direct and indirect effects of the variables on anxiety. Mediation analyses were conducted using the bootstrapping technique proposed by Preacher and Hayes (2004). Bootstrapping does not assume a normal distribution; therefore, 5,000 bootstrap samples were used to obtain 95% CIs and test the significance of the indirect effects.

## Results

First, we analyzed the overall picture of the relationships between anxiety, emotional regulation strategies, perceived sources of stress from the COVID-19 outbreak, and the levels of Happiness and Life-satisfaction. A comparison of the variables’ medians with a median of a normal distribution in the Likert scale range was done to demonstrate the levels of these variables during the COVID-19 pandemic.

At the level of bivariate correlation, Happiness and Life-satisfaction demonstrated a strong correlation (r = 0.62, p < 0.05) (*Table 1*). Happiness, as well as Life-satisfaction, were negatively associated with Anxiety (r = –0.40 for Happiness, r = –0.34 for Life Satisfaction, p < 0.01). Happiness and two perceived sources of stress variables had negative associations: Stress from Pandemic Outcomes (r = –0.11, p < 0.05) and Stress from Everyday Life Restrictions (r = –0.15, p < 0.01). Life-satisfaction demonstrated the same correlations with perceived sources of stress variables (Stress from Pandemic Outcomes (r = –0.10, p < 0.05), and Stress from Everyday Life Restrictions (r = –0.16, p < 0.01)).

**Table 1:** Means, standard deviations, and correlations

Variable	*M*	*SD*	1	2	3	4	5	6	7	8	9
1. Happiness	4.79	1.15									
2. Satisfaction Life	4.14	1.29	0.62**								
3. Anxiety	35.8	0.41	–0.40**	–0.34**							
4. reappraisal Cognitive	5.01	1.05	0.30**	0.25**	–0.18**						
5. suppression Emotional	3.40	1.18	–0.11**	–0.02	0.06	0.16**					
6. Fear Stress of Infection from	2.92	0.90	–0.05	–0.03	0.30**	–0.03	0.06				
7. Virus Stress Spread from	4.28	0.87	–0.07	–0.01	0.21**	0.03	0.00	0.50**			
8. Stress from Pandemic Outcomes	3.36	0.75	–0.11*	–0.10*	–0.30**	–0.01	0.09*	0.40**	0.47**		
9. Stress from Everyday Life Restriction	4.20	0.96	–0.15**	–0.16**	0.31**	0.02	0.10*	0.25**	0.27**	0.34**	
10. Stress from Places and Transport	3.58	1.26	–0.01	–0.01	0.21**	0.12**	–0.02	0.51**	0.49**	0.42**	0.28**

Note: * indicates p < .05; ** indicates p < .01.

Happiness correlated with both emotional regulation strategies; positively with Cognitive Reappraisal (r = 0.30, p < 0.01) and negatively with Emotional Suppression (r = –0.11, p < 0.01). Life-satisfaction only had a positive correlation with Cognitive Reappraisal (r = 0.25, p < 0.01).

Anxiety negatively correlated with Cognitive Reappraisal (r = –0.18, p < 0.01), but not with Emotional Suppression. Anxiety was positively associated with all perceived sources of stress from the COVID-19 outbreak variables (r = 0.21:0.38, p < 0.01). Cognitive Reappraisal positively correlated only with Stress from Places and Transport (r = 0.12, p < .05), and Emotional Suppression with Stress from Pandemic Outcomes (r = 0.09, p < 0.05) and Stress from Everyday Life Restrictions (r = 0.10, p < 0.05). Anxiety negatively correlated with Cognitive Reappraisal (r = –0.18, p < 0.01) but not Emotional Suppression.

Next, two hierarchical regression analyses were performed. (See *Table 2*) Happiness and Life-satisfaction were the dependent variables in each regression. First, we added demographic variables (Gender, Age) and Anxiety; then the perceived sources of stress variables were added in Step 2. Finally, emotional regulation strategies were included in the model.

**Table 2:** Regression results for Happiness and Life-satisfaction

Predictor	Happiness		Live Satisfaction	
	b	beta	b	beta
(Intercept)	6.14**		5.31**	
Gender	0.27*	0.10*	0.45**	0.15**
Age	0.01	0.07	0.00	0.00
Anxiety	–1.13**	–0.40**	–1.11**	–0.35**
Regression result for Step 1	*R^2^* = .182 **		*R^2^* = .140 **	
(Intercept)	6.26**		5.33**	
Gender	0.27*	0.10*	0.45**	0.15**
Age	0.01	0.06	0.00	0.00
Anxiety	–1.16**	–0.41**	–1.11**	–0.35**
Stress from Fear of Infection	0.10	0.08	0.10	0.07
Stress fromVirus Spread	–0.05	–0.03	0.07	0.04
Stress from Pandemic Outcomes	–0.01	–0.01	–0.05	–0.03
Stress from Everyday Life Restriction	–0.06	–0.05	–0.12*	–0.09*
Stress from Places and Transport	0.04	0.05	0.02	0.02
Regression results for Step 2	*R^2^* = .192**, Δ*^R2^* = .009		*R^2^* = .153**, Δ*^R2^* = .013	
(Intercept)	5.40**		4.26**	
Gender	0.15	0.06	0.43**	0.14**
Age	0.00	0.03	–0.00	–0.03
Anxiety	–1.02**	–0.36**	–0.99**	–0.31**
Stress from Fear of Infection	0.13*	0.10*	0.11	0.08
Stress fromVirus Spread	–0.06	–0.05	0.06	0.04
Stress from Pandemic Outcomes	0.02	0.01	–0.04	–0.03
Stress from Everyday Life Restriction	–0.07	–0.05	–0.13*	–0.10*
Stress from Places and Transport	0.00	0.01	–0.01	–0.01
Cognitive reappraisal	0.27**	0.24**	0.22**	0.18**
Emotional suppression	–0.11**	–0.11**	0.01	0.01
Regression results for Step 3	*R^2^* = .245**, Δ*^R2^* = .054**		*R^2^* = .183**, Δ*^R2^* = .030**	

*Note. b represents unstandardized regression weights; beta indicates the standardized regression weights.* *Gender was coded as follows: Male = 1; Female = 2;* * indicates p < .05; ** indicates p < .01.

In the final step, the analysis was found to be statistically significant (F (10 580) = 18.78; p < 0.001). This regression accounted for 24.5% of the variability for Happiness, with four significant predictors. As seen in *Table 2*, Anxiety remained a consistent negative predictor of Happiness from Step 1 to Step 3. Among the perceived sources of stress variables, Stress from Fear of Infection was revealed as a significant predictor for Happiness. Both emotional regulation strategies (Cognitive Reappraisal and Emotional Suppression), added at Step 3, were significant predictors and the explained variance of the model increased (R2 = 0.245**; ΔR2 = 0.054**). Anxiety was the strongest predictor for Happiness, and an increase in Anxiety lowered Happiness even after controlling for gender, age, perceived sources of stress variables, and emotional regulation strategies. The second predictor which decreased Happiness was Emotional Suppression. Cognitive Reappraisal and Stress from Fear of Infection led to an increase in Happiness.

The regression model for Life-satisfaction (*Table 2*) in the final step explained 18.3% of variability (F (10 580) = 12.96; p < 0.001) with three significant predictors; *i.e.,* Anxiety (β = –0.31, p < 0.01), Cognitive Reappraisal (β = 0.18, p < 0.01), and Stress from Everyday Life Restrictions (β = –0.10, p < 0.05). Among the psychological variables, Anxiety was the main predictor for Life-satisfaction in all regression analysis steps with the highest regression weights. Cognitive Reappraisal, but not Emotional Suppression, was revealed as the next significant predictor, and Stress from Everyday Life Restriction had a smaller effect on the variance of Life-satisfaction. It should be mentioned that gender remained a significant predictor at all steps of analysis. Thus, Life-satisfaction, as predicted by Anxiety, Cognitive Reappraisal and Stress from Everyday Life Restrictions, differed according to the gender of the respondents.

### Mediation analysis

The indirect effect of emotional regulation strategies on Happiness and Life-satisfaction was tested using a bootstrapping technique proposed by Preacher and Hayes (2004) with 5,000 bootstrap samples. An indirect effect’s significance is indicated if the 95% confidence interval (CI) does not include zero. To test the hypotheses, two different mediation analyses involving emotional regulation strategies as mediators were conducted.

The indirect effect of Cognitive Reappraisal on Happiness was close to being significant (b = −0.13, SE = 0.04, CI = −0.214, −0.064), and the indirect effects of Emotional Suppression on Happiness were not significant. The same was found for Life-satisfaction. Cognitive Reappraisal’s indirect effect was close to being significant (b = −0.12, SE = 0.04, CI = −0.19: −0.05), and no indirect effect of Emotional Suppression was found. Emotional strategies did not mediate the relationship between Anxiety, and Happiness and Life-satisfaction in this study.

## Discussion

Happiness and Life-satisfaction had the same patterns of associations with other study variables. The fact that anxiety and the perception of the environment as stressful were related to lower levels of Happiness and Life-satisfaction in our study is not surprising. The increase of anxiety and COVID-19-related stress might result from the virus prevention measures’ influence on social and everyday life, including both people’s personal and the overall economic situation. Some previous findings corroborated these associations between anxiety and ill-being, depression, and poor sleep quality (Huang & Zhao, 2020; Zhao et al., 2021b; Pervichko et al., 2022).

We may consider the detrimental role of anxiety as a predictor of Happiness and Life-satisfaction. Anxiety, which accounted for more variance than the other characteristics, explained why some people were more stressed by the pandemic (H1). Therefore, anxiety not only played a negative role but also worked as an enhancer that leads to greater sensitivity to one’s health during a stressful event. In this way, we can speculate that anxiety intensifies the perception of all environments as stressful. This assumption was partially supported by the results of recent research on perceived stress in the world due to the COVID-19 pandemic (Gamonal-Limcaoco et al., 2021), and the fact that high levels of general anxiety might provoke a more severe anxious response to the spread of diseases (Sebri et al., 2021, Blakey & Abramowitz, 2017).

The COVID-19-related perceived risk features had significant but statistically small effects on Happiness and Life-satisfaction (H2). Surprisingly, Stress from Fear of Infection has a relatively small but positive impact on Happiness. Looking at the associations between Stress from Fear of Infection and Anxiety, we may speculate that the incremental effect of Stress from Fear of Infection showed up because of this link. We can suggest a different explanation for the relationship between Stress from Fear of Infection and Happiness. Stress from Fear of Infection increases worries, nervousness, and anxiety, leading to an adaptive strategy to cope with these adverse conditions and to developing cognitive reappraisal. As a result, against the background of the threat of COVID-19 infection, one’s existing lifestyle and achievements begin to be perceived as something valuable. This conclusion could account for a rise in the level of Happiness during the COVID-19 pandemic, but this hypothesis needs additional study and analysis.

Stress from Everyday Life Restrictions had a small effect on Life-satisfaction. According to the result of this study, the limitation of sports activity, social meetings, and the usual course of life lowered subjective well-being. The same results were found in a study of Chinese students, whose results postulated that stress from the pandemic effects on daily life and the delay of academic activities are positively associated with anxiety symptoms and provoke negative emotions (Cao et al., 2020).

Our findings demonstrate that emotional regulation strategies differ in their associations according to the perceived sources of COVID-19 stress. The link between Cognitive Reappraisal, Emotional Suppression, and different pandemic fears may indicate the possible use of emotional regulation strategies to cope with various sources of stress. For example, according to previous studies, before the COVID-19 threat, it was found that Cognitive Reappraisal lowers the emotional reaction to a negative stimulus (Olatunji et al., 2017). However, during the pandemic, this link changed. The reality of infection and its awareness might influence the view of public places as unsafe, which explains a positive relationship between Cognitive Reappraisal and Stress from Places and Transportation.

Emotional regulation strategies had different impacts on Happiness and Life-satisfaction. Previous studies have shown the significantly different roles of adaptive and dysfunctional coping strategies for decreasing or increasing negative emotions during the pandemic (Cincidda et al., 2021). In particular, Restubog et al. (2020) have proven that Cognitive Reappraisal as an adaptive strategy reduces the adverse effects of negative emotions and enhances well-being, and our results confirmed that (H3).

Concerning Life-satisfaction, only Cognitive Reappraisal as an emotional regulation strategy was revealed as a predictor. Many studies consistently prove that adaptive emotional regulation strategies (cognitive reappraisal) are advantageous in reducing symptoms of anxiety (Malikin, et al., 2020; Sebri et al., 2021). People who use cognitive reappraisal negotiate stressful situations by taking an optimistic attitude, reinterpreting what they consider stressful, and making active efforts to avoid bad moods, which should be a protective factor of psychological problems throughout the pandemic situation.

Conversely, the use of maladaptive emotional regulation strategies (emotional suppression) may play a detrimental role in Happiness and Life-satisfaction. Emotional suppression manages the expression of sadness or anxiety, increasing the intensity of those feelings (Gross & John, 2003; Sebri et al., 2021, Dryman & Heimberg, 2018). However, individuals might handle challenging or threatening events successfully through an effective reduction of negative emotions. Current results corroborate previous data about emotional suppression’s impact on emotional experience, social functioning, and overall well-being (Gubler et al., 2021, Dryman & Heimberg, 2018).

Life-satisfaction during the lockdown period was affected by psychological factors such as perceived stress, anxiety, and emotional regulation, and it varied by gender. This finding is consistent with a recent study, which found that women have higher life satisfaction across all social, age, and geographic groups (Joshanloo & Jovanović, 2020), although during the first wave of the pandemic they could demonstrate a higher level of anxiety, avoidance, and hyperarousal (Brivio et al., 2021).

## Conclusion

The point of this study was to explore the effects of anxiety and perceived stress from the COVID-19 pandemic, as well as emotional regulation strategies, during the initial weeks of the lockdown and social isolation.

Psychological health decreased during the COVID-19 outbreak, so researching the mechanisms for coping and regulating emotion is an important task. We identified that high levels of anxiety might be an explanation for the lower level of Happiness and Life-satisfaction during the lockdown period.

Emotional regulation strategies differed in their impacts on well-being. Cognitive reappraisal as an adaptive strategy for dealing with worries and nervousness helped to boost Happiness and Life-satisfaction. In contrast, emotional suppression intensified the feeling of dissatisfaction with life.

We have little evidence that perceived stress from fear of infection would affect Happiness, and that stress from restrictions on everyday life would affect Life-satisfaction. Because the data collection occurred immediately after the lockdown, we might suppose that other sources of stress remained unperceived for some time. We might also propose that anxiety commuted the stress from different sources and revealed it as an overall state that affects well-being. We might speculate that during the first weeks of the lockdown, individual emotional states exceeded the cognitive evaluation and behavioral changes that led to worry and nervousness about the pandemic, and apprehension of disease. Through the early days of the COVID-19 lockdown, the restrictive measures and the virus’s perceived danger threatened societal normalcy, stability, and well-being, revealing anxiety as a prevalent factor in contrast to a rational understanding of potential risks.

The lessons acquired from the pandemic have demonstrated that psychological support should be focused not just on patients and confined individuals, but also on the broader public, to promote psychological well-being. Understanding the cognitive aspects of emotional regulation might pave the way for the development of psychological prevention to avert or mitigate the detrimental effects of pandemics on well-being. Measuring perceived sources of stress during a pandemic might aid future research in evaluating the impact of anxiety-producing events in various locales and in clearly identifying the areas for preventative action.

## Limitations

This study had several limitations. First, it is difficult to make causal inferences despite the data and relevant analyses because of the study design. Second, due to the study’s reliance on a web-based survey and the snowball sampling of participants, the possibility of selection bias must be considered. Third, participants were given the questionnaires in the same order each time, and the probability of the impact of one test’s responses on the next test’s responses was not taken into account. Fourth, it was impossible to assess the participants’ pre-COVID-19 societal psychological characteristics. Fifth, the high rate of female participants and the prevalence of participants from one region may have hindered the validity of the obtained results. These limitations suggest that applying our findings to the general population should be done with care.
